# Exploratory CSF proteomics biomarker outcomes of the the phase 2 clinical trial shine to assess the effects of CT1812 in Alzheimer’s patients

**DOI:** 10.1002/alz.095770

**Published:** 2025-01-09

**Authors:** Valentina Di Caro, Britney N Lizama, Eunah Cho, Duc M Duong, Kiran Pandey, Kaj Blennow, Henrik Zetterberg, Allan I. Levey, Charlotte Teunissen, Anthony O Caggiano, Nicholas T Seyfried, Mary E. Hamby

**Affiliations:** ^1^ Cognition Therapeutics, Inc, Pittsburgh, PA USA; ^2^ Emory University School of Medicine, Atlanta, GA USA; ^3^ Emtherapro, Atlanta, GA USA; ^4^ University of Gotenborg, Gothenburg Sweden; ^5^ University of Gothenburg, Mölndal, Gothenburg Sweden; ^6^ Neurochemistry Laboratory, Amsterdam UMC location VUmc, Amsterdam Netherlands; ^7^ Cognition Therapeutics, Purchase, NY USA

## Abstract

**Background:**

SHINE (NCT03507790, COG0201) is a Phase 2 randomized, double‐blind, placebo‐controlled 6‐month trial, conducted to study the effect of the sigma‐2 receptor (S2R) modulator CT1812 in patients with Alzheimer’s disease (AD). An unbiased assessment of CSF proteomes from the patients that completed the SHINE trial was performed to identify pharmacodynamic (PD) biomarkers of target/pathway engagement and disease modification for CT1812.

**Method:**

Tandem‐mass tag mass spectrometry (TMT‐MS) CSF proteomics was performed on baseline and end of study samples from an analysis of SHINE Part A and B to test the effects of two doses (100 mg, 300 mg; given orally, once daily) of CT1812 compared to placebo in mild to moderate AD patients. Change from baseline was calculated for each participant (N = 47), and differential abundance analysis (CT1812 vs placebo) was performed to assess treatment effects followed by Brain network mapping, Gene Ontology, and pathway analyses using STRING and Metacore (p≤0.1, p≤0.05). Pearson correlation analyses across levels of proteomic proteins and AD core biomarkers were also performed.

**Result:**

In the interim analysis of the first 24 patients (SHINE‐A), differential expression analyses identified proteins altered (p≤0.1 and p≤0.05) in CT1812 vs placebo CSF, and hierarchical clustering demonstrated stratification of patients by treatment. Comparisons to reference standards showed proteins disrupted in or genetically linked to AD that were impacted by CT1812. Proteins dysregulated in AD were found to be normalized with CT1812 treatment (i.e., CLU) and biomarkers of pathway engagement were identified (i.e., APP, NPC1). Brain network mapping and pathway analysis identified statistically significant biological processes altered by CT1812, including those related to synapses, lipoprotein and amyloid beta biology, and neuroinflammation. Analyses of SHINE‐A and ‐B combined (N = 47) will be presented at this meeting.

**Conclusion:**

CSF biomarker findings of the completed SHINE trial extend beyond that previously identified in the interim Part A analysis and shed light on potential biological proteins and/or pathways affected by CT1812. Pharmacodynamic biomarkers of CT1812 pathway engagement and disease modification were identified. Future investigation is warranted to determine if findings replicate in independent cohorts of AD patients.

